# Impact of Integrase Inhibitors on Cardiovascular Disease Events in People With Human Immunodeficiency Virus Starting Antiretroviral Therapy

**DOI:** 10.1093/cid/ciad286

**Published:** 2023-05-09

**Authors:** Bernard Surial, Frédérique Chammartin, José Damas, Alexandra Calmy, David Haerry, Marcel Stöckle, Patrick Schmid, Enos Bernasconi, Christoph A Fux, Philip E Tarr, Huldrych F Günthard, Gilles Wandeler, Andri Rauch, the Swiss HIV Cohort Study, I Abela, K Aebi-Popp, A Anagnostopoulos, M Battegay, E Bernasconi, D L Braun, H C Bucher, A Calmy, M Cavassini, A Ciuffi, G Dollenmaier, M Egger, L Elzi, J Fehr, J Fellay, H Furrer, C A Fux, H F Günthard, A Hachfeld, D Haerry, B Hasse, H H Hirsch, M Hoffmann, I Hösli, M Huber, D Jackson-Perry, C R Kahlert, L Kaiser, O Keiser, T Klimkait, R D Kouyos, H Kovari, K Kusejko, N Labhardt, K Leuzinger, Tejada B Martinez de, C Marzolini, K J Metzner, N Müller, J Nemeth, D Nicca, J Notter, P Paioni, G Pantaleo, M Perreau, A Rauch, L Salazar-Vizcaya, P Schmid, R Speck, M Stöckle, P Tarr, A Trkola, G Wandeler, M Weisser, S Yerly

**Affiliations:** Department of Infectious Diseases, Inselspital, Bern University Hospital, University of Bern, Bern, Switzerland; Division of Clinical Epidemiology, Department of Clinical Research, University Hospital Basel, University of Basel, Basel, Switzerland; Division of Infectious Diseases, University Hospital of Lausanne, University of Lausanne, Lausanne, Switzerland; Division of Infectious Diseases, Geneva University Hospital, University of Geneva, Geneva, Switzerland; Chair Positive Council, Zurich, Switzerland; Division of Infectious Diseases and Hospital Epidemiology, University Hospital Basel, University of Basel, Basel, Switzerland; Division of Infectious Diseases, Cantonal Hospital of St Gallen, St Gallen, Switzerland; Division of Infectious Diseases, Ente Ospedaliero Cantonale Lugano, University of Geneva and University of Southern Switzerland, Lugano, Switzerland; Division of Infectious Diseases, Cantonal Hospital of Aarau, Aarau, Switzerland; Department of Medicine and Division of Infectious Diseases and Hospital Epidemiology, Kantonsspital Baselland, University of Basel, Bruderholz, Switzerland; Department of Infectious Diseases and Hospital Epidemiology, University Hospital Zurich, University of Zurich, Zurich, Switzerland; Institute of Medical Virology, University of Zurich, Zurich, Switzerland; Department of Infectious Diseases, Inselspital, Bern University Hospital, University of Bern, Bern, Switzerland; Department of Infectious Diseases, Inselspital, Bern University Hospital, University of Bern, Bern, Switzerland

**Keywords:** antiretroviral therapy, treatment-naïve, myocardial infarction, stroke, integrase strand transfer inhibitor

## Abstract

**Background:**

Integrase strand transfer inhibitors (INSTIs) have been associated with an increased risk for cardiovascular disease (CVD) events. We investigated the impact of starting INSTI-based antiretroviral therapy (ART) on CVD events among treatment-naïve people with human immunodeficiency virus using a target trial framework, which reduces the potential for confounding and selection bias.

**Methods:**

We included Swiss HIV Cohort Study participants who were ART-naïve after May 2008, when INSTIs became available in Switzerland. Individuals were categorized according to their first ART regimen (INSTI vs other ART) and were followed from ART start until the first of CVD event (myocardial infarction, stroke, or invasive cardiovascular procedure), loss to follow-up, death, or last cohort visit. We calculated hazard ratios and risk differences using pooled logistic regression models with inverse probability of treatment and censoring weights.

**Results:**

Of 5362 participants (median age 38 years, 21% women, 15% of African origin), 1837 (34.3%) started INSTI-based ART, and 3525 (65.7%) started other ART. Within 4.9 years (interquartile range, 2.4–7.4), 116 CVD events occurred. Starting INSTI-based ART was not associated with an increased risk for CVD events (adjusted hazard ratio, 0.80; 95% confidence interval [CI], .46–1.39). Adjusted risk differences between individuals who started INSTIs and those who started other ART were −0.17% (95% CI, −.37 to .19) after 1 year, −0.61% (−1.54 to 0.22) after 5 years, and −0.71% (−2.16 to 0.94) after 8 years.

**Conclusions:**

In this target trial emulation, we found no difference in short- or long-term risk for CVD events between treatment-naïve people with human immunodeficiency virus who started INSTI-based ART and those on other ART.

## Teaser

In treatment-naïve people with HIV, starting antiretroviral therapy containing integrase strand transfer inhibitors did not lead to an increased risk for cardiovascular disease events in this target trial emulation from the Swiss HIV Cohort Study.

## Graphical Abstract

**Graphical Abstract:**
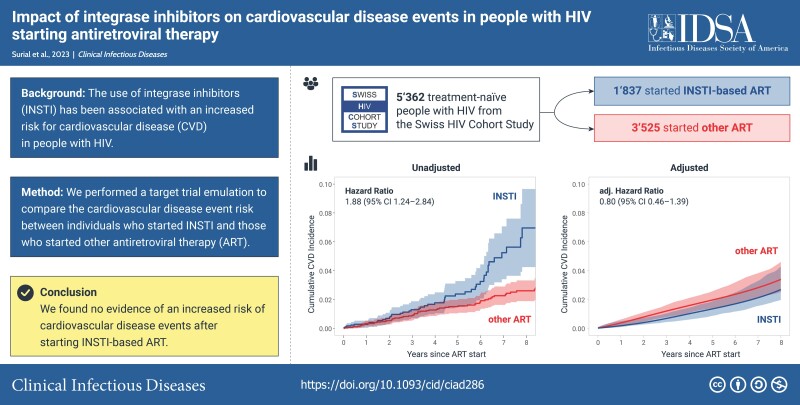
This graphical abstract is also available at Tidbit: https://tidbitapp.io/tidbits/impact-of-integrase-inhibitors-on-cardiovascular-disease-events-in-people-with-hiv-starting-antiretroviral-therapy-2cc8a06e-dbe6-4aea-a856-cef20beefd00

**(See the Editorial Commentary by Costagliola on pages 738–9.)**

## INTRODUCTION

Integrase strand transfer inhibitors (INSTIs) have become the mainstay of modern antiretroviral treatment (ART). Compared with nonnucleoside reverse transcriptase inhibitors (NNRTIs) or protease inhibitors (PIs), INSTIs have improved virological efficacy, better tolerability, and a lower potential for drug–drug interactions (DDI) [1–3]. In addition, treatment-emergent resistance has become exceedingly rare because of the high genetic barrier to resistance of dolutegravir and bictegravir [4, 5]. Therefore, INSTI-based regimens are the preferred recommended initial ART of all major human immunodeficiency virus (HIV) treatment guidelines [6–10], and dolutegravir-based first-line therapy is currently being rolled out programmatically for people with HIV (PWH) worldwide [11, 12].

However, concerns about cardiometabolic complications have emerged in recent years. INSTI-based ART is associated with weight increases among treatment-naïve and treatment-experienced individuals [13–15], and an increased incidence of arterial hypertension and diabetes has been observed in people newly starting INSTI-based ART [16, 17]. In addition, a recent analysis of a large collaboration of HIV cohorts in Europe and Australia (RESPOND) found an increased risk for cardiovascular disease (CVD) events with the use of INSTI compared with individuals who did not receive INSTIs [18]. The largest increase was observed within the first 2 years after the initiation of INSTI-based ART, with a return to similar rates in both groups thereafter. Because cardiovascular complications remain a major driver of morbidity and mortality among PWH, the potential contribution of INSTIs to CVD is a major global public health concern [19].

Although arterial hypertension and obesity can contribute to the development of CVD over time, the development of atherosclerosis takes time and is usually not rapidly reversible. Given these pathophysiological mechanisms, the early and transient increase in CVD risk observed in the RESPOND study is unexpected. Because the study analyzed treatment-naïve and treatment-experienced individuals together and applied different inclusion criteria for individuals with and without INSTI-based ART, methodological issues such as confounding by indication, immortal time, and selection bias may have contributed to the findings.

The target trial framework helps reduce the potential for bias by specifically aligning the start of follow-up (time 0), eligibility, and the treatment assignment using observational data [20]. In this study, we aimed to emulate a target trial to estimate the effects of starting INSTI-based ART compared with ART without INSTIs on CVD events within the Swiss HIV Cohort Study (SHCS).

## METHODS

### Study Design

We emulated a target trial in which treatment-naïve PWH were assigned to start either INSTI-based or other ART as their first treatment regimen. We used data from the SHCS (www.shcs.ch), an ongoing prospective cohort established in 1988, which includes close to 80% of individuals who receive ART in Switzerland. Sociodemographic, clinical, and behavioral data as well as laboratory values are prospectively recorded at registration and every 6 months thereafter using standardized protocols (http://shcs.ch/292-instructions). Information on the full ART history and comedications is collected at every cohort visit using an online data entry tool [21]. All centers’ local ethical committees approved the cohort study and all patients provided written informed consent. Patient representatives were involved in the planning of the study, interpretation of the results, and writing of the manuscript. The reporting of the study follows the Strengthening the Reporting of Observational Studies in Epidemiology guidelines [22].

### Eligibility Criteria and Treatment Strategies

We considered all cohort participants who were HIV treatment-naïve after 1 May 2008 (when the first INSTI raltegravir was licensed in Switzerland) and who initiated ART thereafter. Individuals who started unknown ART, those who initiated ART including maraviroc or enfuvirtide, and individuals who had an HIV RNA at treatment start <50 cp/mL were excluded because those individuals may not have been treatment naïve. INSTI starters included all participants who received any INSTI-containing ART as first treatment, regardless of whether other ART components (eg, NNRTI, PI) were coadministered.

### Follow-up, Outcomes, and Definitions

Time 0 was defined as date of first ART start, and individuals were followed until the first CVD event, loss to follow-up, death, or last cohort visit (database closure on 30 September 2022). Individuals in the INSTI group who stopped INSTIs during follow-up were censored at that time, and those who received an initial ART without INSTIs were censored when they switched to an INSTI-based therapy (artificial censoring). The outcome of interest was the time to the first CVD event, which included myocardial infarction, stroke, and invasive cardiovascular procedures. CVD events were collected using a dedicated case report form and validated from a senior HIV physician at the cohort site. In addition, central validation was performed using a standardized algorithm blinded to the ART history of the patient [23].

### Statistical Analysis

We emulated a target trial to compare the risk of CVD events between treatment-naïve PWH who started INSTIs and those who started other ART. The time to CVD event was analyzed using pooled logistic regression models with inverse probability weighting to adjust for confounding, artificial censoring, and loss to follow-up. Stabilized treatment weights were estimated by fitting a logistic regression model with treatment allocation as the outcome, and the following time-fixed confounders as covariates (all evaluated at or before ART start): calendar year, age, sex, ethnicity (Caucasian, African origin, and other), HIV transmission group (men who have sex with men, heterosexual contacts, people who inject drugs, and other), highest education (high-level, basic, and no professional education), CD4 at ART start (≥500 cells/µL, 350–499 cells/µL, 200–349 cells/µL, < 200 cells/µL), HIV DNA at ART start (50–199 cp/mL, 200–100 000 cp/mL, > 100 000 cp/mL), personal and family history of CVD, body mass index ( < 18.5 kg/m^2^, 18.5–24.9 kg/m^2^, 25–29.9 kg/m^2^, ≥ 30 kg/m^2^), arterial hypertension, diabetes, total cholesterol, high-density lipoprotein, renal function (estimated glomerular filtration rate [eGFR] ≥ 90 mL/min, 60–89 mL/min, or <60 mL/min), current use of antiplatelet or lipid-lowering drugs, and current use of abacavir (ABC) or tenofovir alafenamide (TAF). Arterial hypertension was defined as a systolic blood pressure >140 mm Hg or a diastolic blood pressure >90 mm Hg on 2 cohort visits or receiving antihypertensive drugs. Diabetes was defined as a hemoglobin A1c level of 6.5% or greater or receiving antidiabetic medication. The eGFR was calculated using the Chronic Kidney Disease-Epidemiology Collaboration formula [24]. Follow-up time was included in the model using restricted cubic splines with 3 knots. Similarly, we fitted a separate pooled logistic regression model in a person-time format, with loss to follow-up as the outcome, and time-fixed values for calendar year at ART start, age, sex, ethnicity, HIV transmission group, highest education, arterial hypertension, diabetes, renal function, total cholesterol, high-density lipoprotein, and body mass index. In addition, time-varying values for smoking, CD4 cell count, HIV RNA, and use of ABC, TAF, lipid-lowering, or antiplatelet drug use were included in the model. CVD risk factors were not included as time-varying covariates because they may lie on the causal path between the exposure and the outcome, and therefore adjusting for those mediating covariates could have led to biased estimates. We fitted a third pooled logistic regression model to estimate weights for artificial censoring, with the same covariates as described in the loss to follow-up model. The loss to follow-up and artificial censoring models were fitted individually for each treatment group (INSTI-based or other ART) as reasons for censoring may have been different between both groups. Finally, we incorporated all weights into a pooled logistic regression model and estimated marginal probabilities of CVD events after 6 months, 1, 2, 5, and 8 years, and calculated risk differences and risk ratios between both groups. We calculated 95% confidence interval (CI) using nonparametric bootstrapping with 200 samples. In addition, we calculated hazard ratios after 8 years for the main and all subgroup and sensitivity analyses using weighted pooled logistic regression models as described previously and used robust standard errors to calculate 95% CI. Because the missing at random assumption may not have held, we did not impute missing data but included a separate missing category for each covariate with missing information. All analyses were performed with R version 4.1.3 [25].

### Subgroup and Sensitivity Analyses

To identify whether treatment effects varied according to patient groups, we performed prespecified subgroup analyses according to sex, age (<65 or ≥65 years), and whether ART included ABC or not. We also performed the following sensitivity analyses to evaluate the robustness of our results: first, we restricted the analysis to individuals who started ART when INSTIs became part of the recommended first-line regimens in the European AIDS Clinical Society Guidelines (November 2011) [6], after which INSTI-based ART was prescribed more broadly in Switzerland. Second, we limited the analysis to individuals without a history of CVD because their risk to develop a subsequent event is higher than the risk of PWH without past CVD events.

## RESULTS

**Figure 1.**
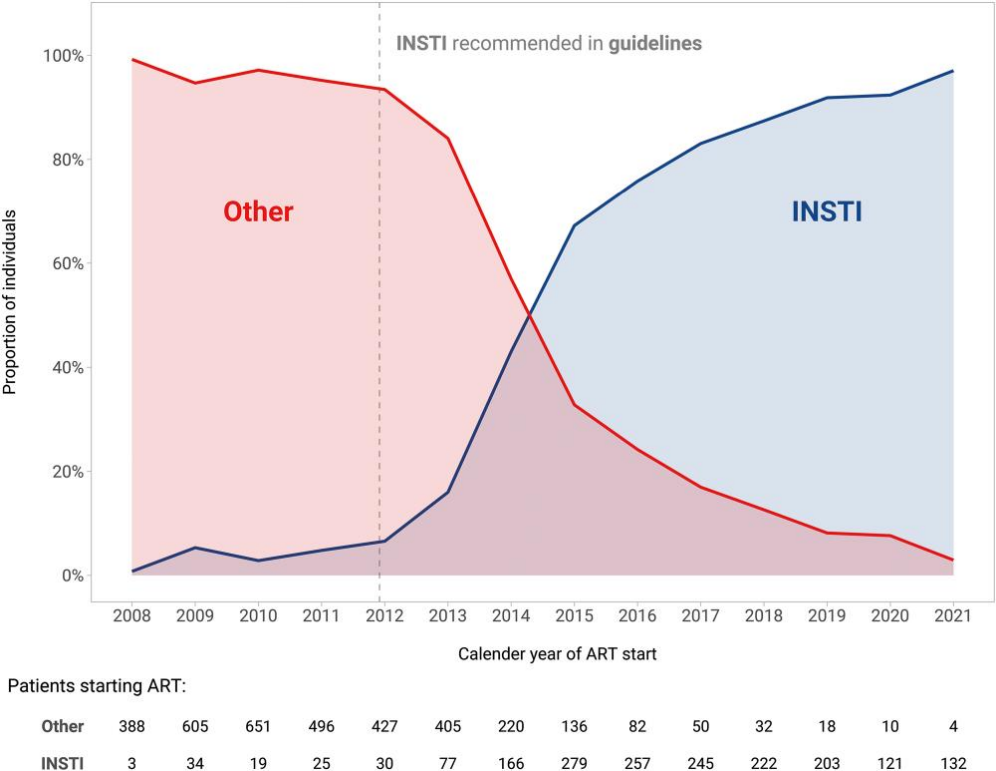
Use of integrase strand transfer inhibitor (INSTI) as initial antiretroviral treatment over time. Representation of the proportion of treatment-naïve individuals who received INSTI-based or other antiretroviral therapy (ART) combinations as first-line treatment in the Swiss HIV Cohort Study (SHCS) over time. After INSTIs were recommended as the preferred initial regimens in major treatment guidelines (vertical dashed line), uptake of INSTIs rapidly increased: in 2021, 96% of individuals starting ART received INSTI compared with 4% who received other ART.

Of 13 767 SHCS participants with available follow-up after May 2008, 6027 started their first ART regimen after that date. After excluding 24 individuals with an unknown ART regimen, 7 individuals who received maraviroc as part of their initial ART regimen, and 634 individuals with an undetectable HIV viral load at ART start, the study population included 5362 PWH (Supplementary Figure 1). The median age was 38 years (interquartile range [IQR], 31–47), 1145 (21%) were women, and 817 (15%) were of African origin. Overall, 1837 (34.3%) started INSTI-containing ART and 3525 (65.7%) started other ART combinations. INSTI uptake was low before the the inclusion of INSTIs as the preferred regimen in European guidelines, and became the predominant ART component after 2015 (Figure 1). The most frequent INSTI initiated was dolutegravir (n = 984, 53%), followed by bictegravir (n = 325, 18%), elvitegravir (n = 298, 16%), and raltegravir (231, 13%). Of individuals who started INSTIs, 152 (8%) additionally received a PI or an NNRTI. Among PWH who started other ART, 1826 (52%) received PI-containing ART, 1595 (45%) received NNRTI-containing ART, and 104 (3%) received a combination thereof. Individuals who started INSTIs were less likely to be women, less likely to be of African origin, and had a higher median CD4 nadir compared with individuals with other ART combinations. Smoking status, history of CVD, and use of antiplatelet agents were similar in both groups, but INSTI starters were more likely to have an eGFR <60 mL/min and to receive abacavir and tenofovir alafenamide (Table 1). Among individuals who started ART after 2012, those who started INSTI-based ART were more likely to have arterial hypertension, diabetes, a history of CVD, and an eGFR below 90 mL/min compared with those who started other ART (Supplementary Table 1). The covariate balance before and after inverse probability weighting is shown in Supplementary Figure 2. Artificial censoring occurred in 111 individuals (6%) who started INSTIs and switched to non-INSTI ART during follow-up and in 2217 individuals (62.9%) who started other ART and switched to INSTI-based therapy during follow-up.

**Table 1.** Characteristics of Study Participants at ART Start

Characteristic	INSTI-based ART, N = 1837	Other ART, N = 3525
Female	291 (16%)	854 (24%)
Median age, y (IQR)	39 (31–50)	38 (31–46)
African origin	199 (11%)	618 (18%)
HIV transmission group		
MSM	1099 (60%)	1846 (52%)
Heterosexual contacts	550 (30%)	1269 (36%)
PWID	74 (4.0%)	196 (5.6%)
Other	114 (6.2%)	214 (6.1%)
Highest education		
High-level education	835 (45%)	1355 (38%)
Basic education	874 (47%)	1915 (55%)
No professional education	100 (5%)	219 (6%)
Missing	28 (1%*)*	36 (1%*)*
AIDS-defining disease	196 (11%)	395 (11%)
Median CD4 nadir, cells/µL (IQR)	330 (188–482)	278 (171–372)
CD4 nadir		
≥500 cells/µL	360 (20%)	300 (8.5%)
350–499 cells/µL	365 (20%)	613 (17%)
200–349 cells/µL	411 (22%)	1’166 (33%)
<200 cells/µL	405 (22%)	907 (26%)
Missing	296 (16%)	539 (15%)
HIV RNA at ART start		
50–199 cp/mL	87 (4.7%)	217 (6.2%)
200–100 000 cp/mL	991 (54%)	1911 (54%)
>100 000 cp/mL	594 (32%)	986 (28%)
Missing	165 (9.0%)	411 (12%)
Year of ART start		
Before 2012	81 (4.4%)	2140 (61%)
2012–2016	809 (44%)	1270 (36%)
after 2016	947 (52%)	115 (3.3%)
History of CVD	28 (1.5%)	44 (1.2%)
Family history of CVD	209 (11%)	352 (10%)
Diabetes	39 (2.1%)	62 (1.8%)
Arterial hypertension	189 (10%)	350 (9.9%)
Renal function (eGFR)		
≥90 mL/min	1203 (65%)	2227 (63%)
60–89 mL/min	365 (20%)	531 (15%)
<60 mL/min	42 (2.3%)	54 (1.5%)
Missing	227 (12%)	713 (20%)
Median eGFR, mL/min (IQR)	104 (90–115)	106 (93–118)
Smoking status		
Current	842 (46%)	1660 (47%)
Never	783 (43%)	1535 (44%)
Past	192 (10%)	322 (9.1%)
Missing	20 (1.1%)	8 (0.2%)
Median BMI, kg/m^2^ (IQR)	23.6 (21.4–26.3)	23.2 (21.2–25.7)
BMI category		
Normal (18.5–24.9 kg/m^2^)	858 (47%)	1624 (46%)
Overweight (25–29.9 kg/m^2^)	374 (20%)	606 (17%)
Obese (≥30 kg/m^2^)	121 (6.6%)	178 (5.0%)
Underweight (<18.5 kg/m^2^)	78 (4.2%)	152 (4.3%)
Missing	406 (22%)	965 (27%)
Use of antiplatelet agent	34 (1.9%)	54 (1.5%)
Use of lipid-lowering drug	48 (2.6%)	62 (1.8%)
Use of abacavir	430 (23%)	411 (12%)
Use of tenofovir alafenamide	730 (40%)	48 (1.4%)

Abbreviations: ART, antiretroviral therapy; BMI, body mass index; CVD, cardiovascular disease; eGFR, estimated glomerular filtration rate; INSTI, integrase strand transfer inhibitor; IQR, interquartile range; MSM, men who have sex with men; PWID, persons who inject drugs.

**Figure 2.**
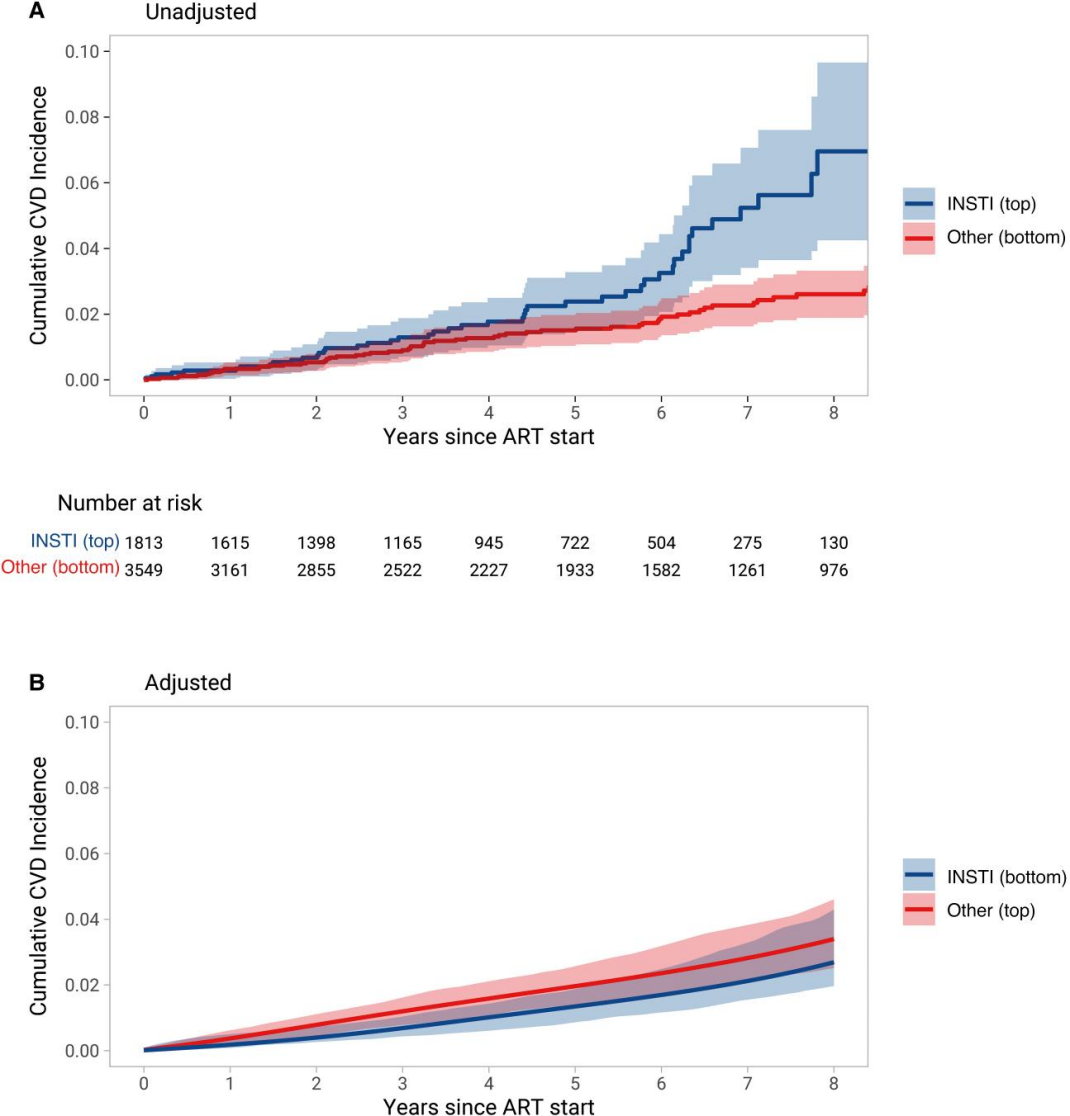
Cumulative incidence of cardiovascular disease (CVD) events after starting antiretroviral therapy. (*A*) The curves represent the unadjusted cumulative incidence of CVD events over time. (*B*) Curves of cumulative CVD incidence, adjusted for confounders and informative censoring (see Methods section for a full list of covariates). Shaded areas represent 95% confidence intervals.

Within a median follow-up of 4.9 years (IQR, 2.4–7.4), 116 CVD events occurred: 37 myocardial infarctions (31.9%), 36 strokes (31.0%), and 43 invasive cardiovascular procedures (37.1%, Supplementary Table 2). The unadjusted incidence rate for CVD events was 6.16 per 1000 person-years (95% CI, 4.64–8.17) for individuals who started INSTIs and 3.54 per 1000 person-years (95% CI, 2.79–4.49) for individuals who started other ART (hazard ratio [HR], 1.88; 95% CI, 1.24–2.84; Figure 2*A*). After adjusting for confounding and informative censoring, the HR comparing individuals who started INSTIs with those who started other ART was 0.80 (95% CI, .46–1.39; Figure 2*B*). Adjusted risk ratios and risk differences between both treatment groups are presented in Table 2. Adjusted risk differences between individuals who started INSTIs and those who started other ART ranged from −0.08% (95% CI, −.20 to .19) after 6 months to −0.71% (95% CI, −2.16 to .94) after 8 years.

**Table 2.** Cardiovascular Disease Event Risks After Starting Antiretroviral Therapy

Time Since ART Start	INSTI		Other ART		Adjusted^a^ Risk Difference	Adjusted^a^ Risk Ratio
	Unadjusted CVD Event Risk	Adjusted^a^ CVD Event Risk	Unadjusted CVD Event Risk	Adjusted^a^ CVD Event Risk		
6 months	0.28%	0.08%	0.12%	0.16%	−0.08% (−0.20 to 0.19)	0.49 (0.11–3.35)
1 year	0.28%	0.17%	0.33%	0.34%	−0.17% (−0.37 to 0.19)	0.49 (0.20–1.86)
2 years	0.68%	0.38%	0.54%	0.75%	−0.37% (−0.72 to 0.09)	0.50 (0.28–1.17)
5 years	2.38%	1.32%	1.51%	1.93%	−0.61% (−1.54 to 0.22)	0.68 (0.37–1.14)
8 years	6.94%	2.64%	2.61%	3.34%	−0.71% (−2.16 to 0.94)	0.79 (0.49–1.34)

Abbreviations: ART, antiretroviral therapy; CVD, cardiovascular disease; INSTI, integrase strand transfer inhibitor.

Adjusted for confounding and informative censoring (refer to Methods for a detailed list of covariates).

**Figure 3.**
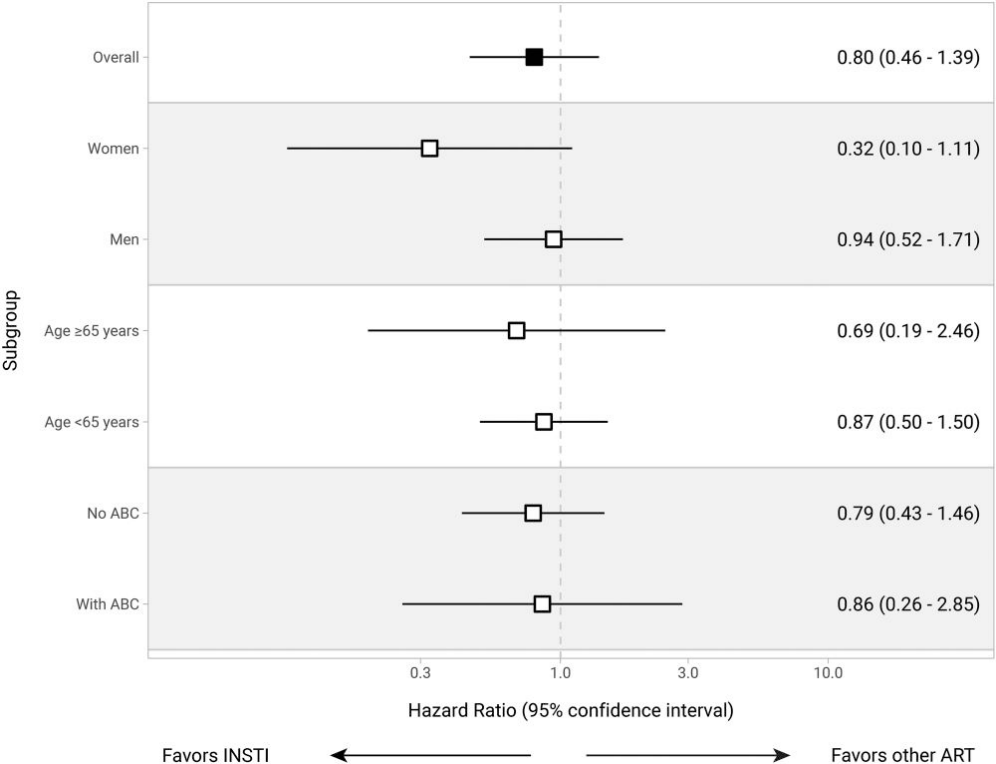
Impact of INSTI on cardiovascular disease risk overall and by prespecified subgroups. The forest plot shows hazard ratios (95% confidence intervals) adjusted for confounders and informative censoring (see Methods section for a full list of covariates). ABC, abacavir; INST, integrase strand transfer inhibitor.

The impact of starting INSTIs compared with other ART was consistent across age groups (≥65 years vs < 65 years, *P* value for interaction = .72) and did not vary according to use of ABC or not (*P* value for interaction = .91). However, the relative risk of CVD associated with starting INSTIs compared with other ART appeared to be lower among women, but this result was not statistically significant (adjusted HR, 0.32; 95% CI, .10–1.11, *P* value for interaction = 0.112; Figure 3). In a sensitivity analysis excluding individuals with a history of CVD events before ART start (n = 72), the adjusted HR for developing a new CVD event with INSTIs compared with other ART was 0.74 (95% CI, .39–1.42; Supplementary Figure 3). When restricting analyses to individuals who started ART after November 2011, when INSTI became part of the recommended initial ART regimens (n = 3216, of whom 1763 started INSTIs and 1453 started other ART), the HR for developing CVD events was similar to the main analysis (adjusted HR, 0.94; 95% CI, .40–2.22; Supplementary Figure 4).

## DISCUSSION

In this nationwide cohort study of 5362 treatment-naïve PWH and a median follow-up of 5 years, we found no difference in risk for developing CVD events between individuals who started an INSTI-based ART and those who started other ART combinations. These findings are reassuring in the context of increasing INSTI use worldwide and support the prioritization of INSTIs as the preferred agents in current international guidelines [6–10].

Our main findings contrast with those from RESPOND, who observed a marked increase in CVD risk between 6 and 24 months after the initiation of an INSTI [18]. Whereas the RESPOND study included both treatment-naïve and treatment-experienced cohort participants, we limited our analysis to individuals who started their first ART regimen during the INSTI era. This approach provided better alignment of time 0 and reduced the potential for immortal time bias [20]. In addition, whereas the target trial framework required us to apply the same selection criteria for both exposure groups, the RESPOND study only included individuals who were not exposed to INSTIs before 2012, which may have introduced bias because of the selective left-censoring of individuals who were exposed to INSTIs.

A previous registry-based study from the United States documented a lower risk for CVD events among 20'000 treatment-naïve PWH who started INSTI-based ART compared with those who started other ART [26]. Differences in outcome assessment and availability of clinical data may explain some of the discrepancies with the present study because O’Halloran et al. used administrative data from Medicaid, whereas the SHCS provides detailed longitudinal data with stringent ascertainment of CVD outcomes. The prespecified subgroup of women appeared to have a lower CVD risk with the use of INSTI in our study, although the CIs were wide and included a null effect. This finding was unexpected, but its interpretation needs to be cautious because it was based on low patient numbers.

Our estimates for the impact on CVD events of starting INSTIs compared with other ART combinations differed markedly between unadjusted and adjusted analyses. In part, these differences may be explained by confounding because individuals who started INSTIs were more likely to be men and to receive abacavir, factors that were previously associated with an increased CVD risk [27, 28]. Furthermore, when examining patient characteristics stratified over time periods, individuals receiving INSTIs after 2012 were older and more likely to have additional CVD risk factors. In addition, a large proportion of individuals who started other ART eventually switched to INSTIs over time. Individuals with comorbidities may have been prioritized to switching to INSTIs because of their lower potential for DDI, leading to selection bias in the form of informative censoring. We addressed both confounding and selection bias by applying the target trial framework and using inverse probability of censoring and treatment weights in our study.

We explored the association between INSTI-based ART and CVD risk using a target trial emulation, a framework that reduces the potential for confounding by indication and selection bias. The study was performed using data from the nationally representative cohort of PWH and showed consistent results across several sensitivity analyses. We relied on long-term follow-up of individuals with and without INSTI-based ART, which allowed us to consider both older and newer generation INSTIs. Furthermore, the SHCS follows a very stringent outcome ascertainment based on prespecified protocols and blinded central validation. However, the relatively low number of CVD events in our study limited the statistical power to detect a difference in CVD risk between groups during each specific time interval and prevented us from performing separate analyses comparing INSTIs with PI or NNRTI starters, as well as evaluating the role of individual INSTI components. In addition, because our study was restricted to treatment-naïve individuals who started ART, our findings cannot be extrapolated to treatment-experienced individuals who switch to an INSTI-based regimen. Dedicated target trial emulations focused on individuals who switch to INSTI-based ART are needed because a simultaneous switch from tenofovir disoproxil fumarate (TDF) to TAF is frequent and may contribute to the CVD risk [29]. Furthermore, although we accounted for unmeasured changes in CVD prevention over time by including calendar year in our models, some residual impact on our findings cannot be excluded. Moreover, the difference in follow-up between both groups could potentially lead to selection bias because individuals who did not experience an event over a longer period may have been healthier and at lower risk for CVD events. However, we accounted for these differences using inverse probability of censoring weighting, which mitigates the impact of differential follow-up time. Finally, even if our results argued against an increase in CVD risk with the use of INSTIs, the duration of observation of our study may have been insufficient to capture a gradual increase in CVD risk mediated through INSTI-associated weight increases and arterial hypertension.

In conclusion, we found no evidence of a difference in CVD event risk between treatment-naïve individuals who started INSTI-based ART and those who started other ART combinations. Importantly, the CVD risk was similar between both groups in the short term as well as in the longer term. Similarly designed studies are needed to evaluate the impact of switching to INSTI-based ART among treatment-experienced individuals to further understand the impact of INSTI on cardiometabolic outcomes.

## Supplementary Data

Supplementary materials are available at *Clinical Infectious Diseases* online. Consisting of data provided by the authors to benefit the reader, the posted materials are not copyedited and are the sole responsibility of the authors, so questions or comments should be addressed to the corresponding author.

## Supplementary Material

ciad286_Supplementary_Data

## Notes

***Author contributions*.** B. S., F. C., H. F. G., G. W., and A.R. conceived and designed the study. B. S. performed the statistical analyses and drafted the initial manuscript. All authors contributed data to the study and to the interpretation of the results and revised the manuscript for substantial intellectual content. All authors read and approved the final manuscript.

***Acknowledgments***. The authors thank all patients, physicians, and nurses associated with the Swiss HIV Cohort Study (SHCS). The members of the SHCS are Abela I, Aebi-Popp K, Anagnostopoulos A, Battegay M, Bernasconi E, Braun DL, Bucher HC, Calmy A, Cavassini M, Ciuffi A, Dollenmaier G, Egger M, Elzi L, Fehr J, Fellay J, Furrer H, Fux CA, Günthard HF (president of the SHCS), Hachfeld A, Haerry D (deputy of “Positive Council”), Hasse B, Hirsch HH, Hoffmann M, Hösli I, Huber M, Jackson-Perry D (patient representatives), Kahlert CR (chairman of the Mother & Child Substudy), Kaiser L, Keiser O, Klimkait T, Kouyos RD, Kovari H, Kusejko K (head of Data Centre), Labhardt N, Leuzinger K, Martinez de Tejada B, Marzolini C, Metzner KJ, Müller N, Nemeth J, Nicca D, Notter J, Paioni P, Pantaleo G, Perreau M, Rauch A (chairman of the Scientific Board), Salazar-Vizcaya L, Schmid P, Speck R, Stöckle M (chairman of the Clinical and Laboratory Committee), Tarr P, Trkola A, Wandeler G, Weisser M, Yerly S.

***Financial support*.** This work was funded by the framework of the SHCS, supported by the Swiss National Science Foundation (SNF grant number 201369, SHCS project number 909), and by the SHCS research foundation. G. W. was supported by a Professorship from the Swiss National Science Foundation (PP00P3_211025). The data were gathered by 5 Swiss University Hospitals, 2 cantonal hospitals, 15 affiliated hospitals, and 36 private physicians (listed in http://www.shcs.ch/180-health-care-providers).
