# Supplementary material for: Impact of Integrase Inhibitors on Cardiovascular Disease Events in People With Human Immunodeficiency Virus Starting Antiretroviral Therapy
Source: Clin Infect Dis. 2023 May 9;77(5):729–37. doi: 10.1093/cid/ciad286 (PMC10495132; doi:10.1093/cid/ciad286)
Supplement: ciad286_Supplementary_Data [file ciad286_supplementary_data.docx]

**Supplementary Material:**

**Impact of integrase inhibitors on cardiovascular disease events in people with HIV starting antiretroviral therapy**

Bernard Surial, Frédérique Chammartin, José Damas, Alexandra Calmy, David Haerry, Marcel Stöckle^,^ Patrick Schmid, Enos Bernasconi, Christoph A. Fux, Philip Tarr, Huldrych F. Günthard, Gilles Wandeler, Andri Rauch, and the Swiss HIV Cohort Study

**Table of contents**

**Figure S1:** Selection of the study population 2

**Figure S2:** Covariate balance 3

**Figure S3:** Sensitivity analysis 4

**Figure S4:** Sensitivity analysis 5

**Table S1:** Patient characteristics across follow-up periods 6

**Table S2:** Distribution of follow-up, outcome events and censoring events 7

**Figure S1** Selection of the study population


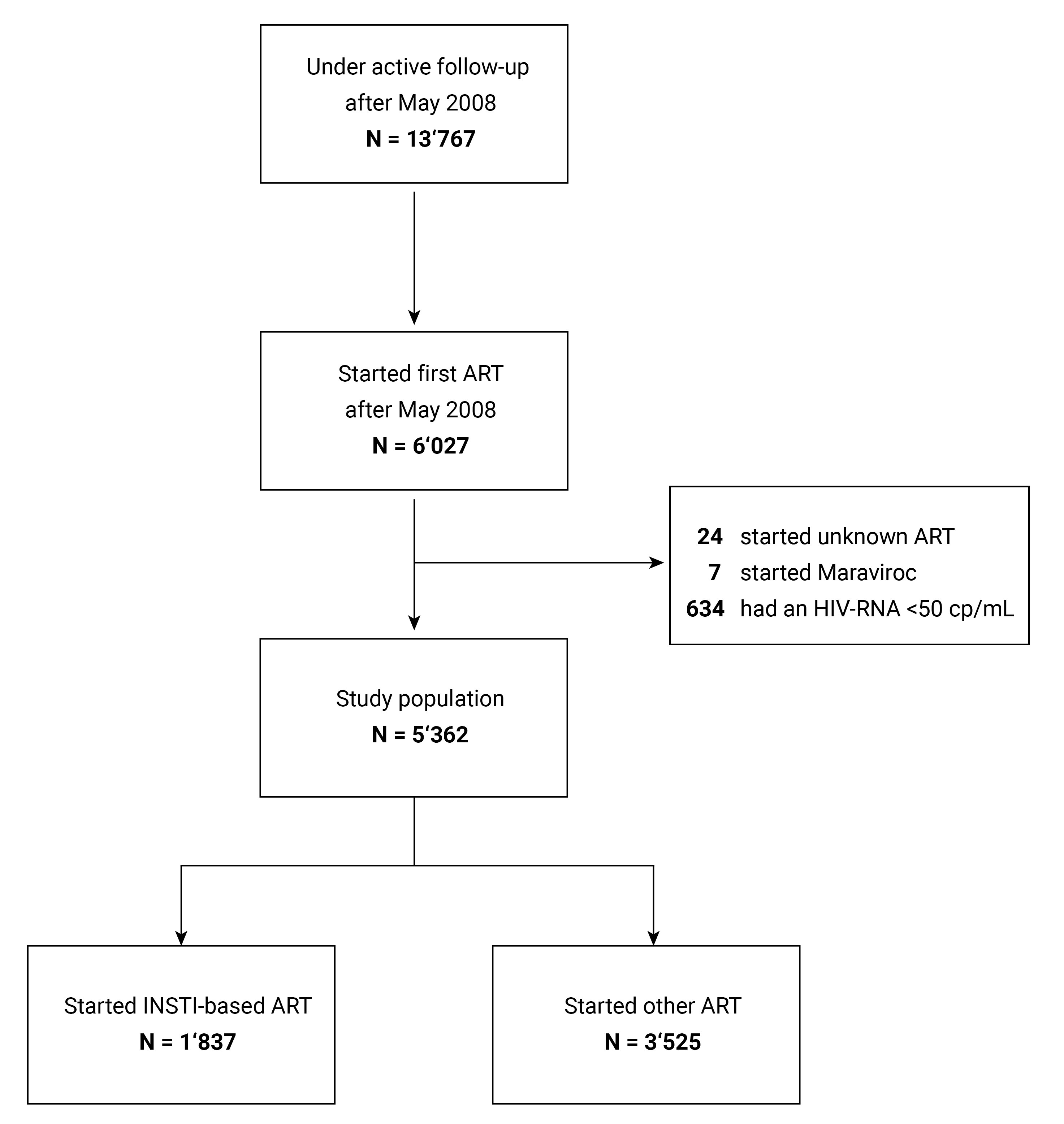


**Figure S2** Covariate balance before (light-grey) and after (black) inverse probability weighting


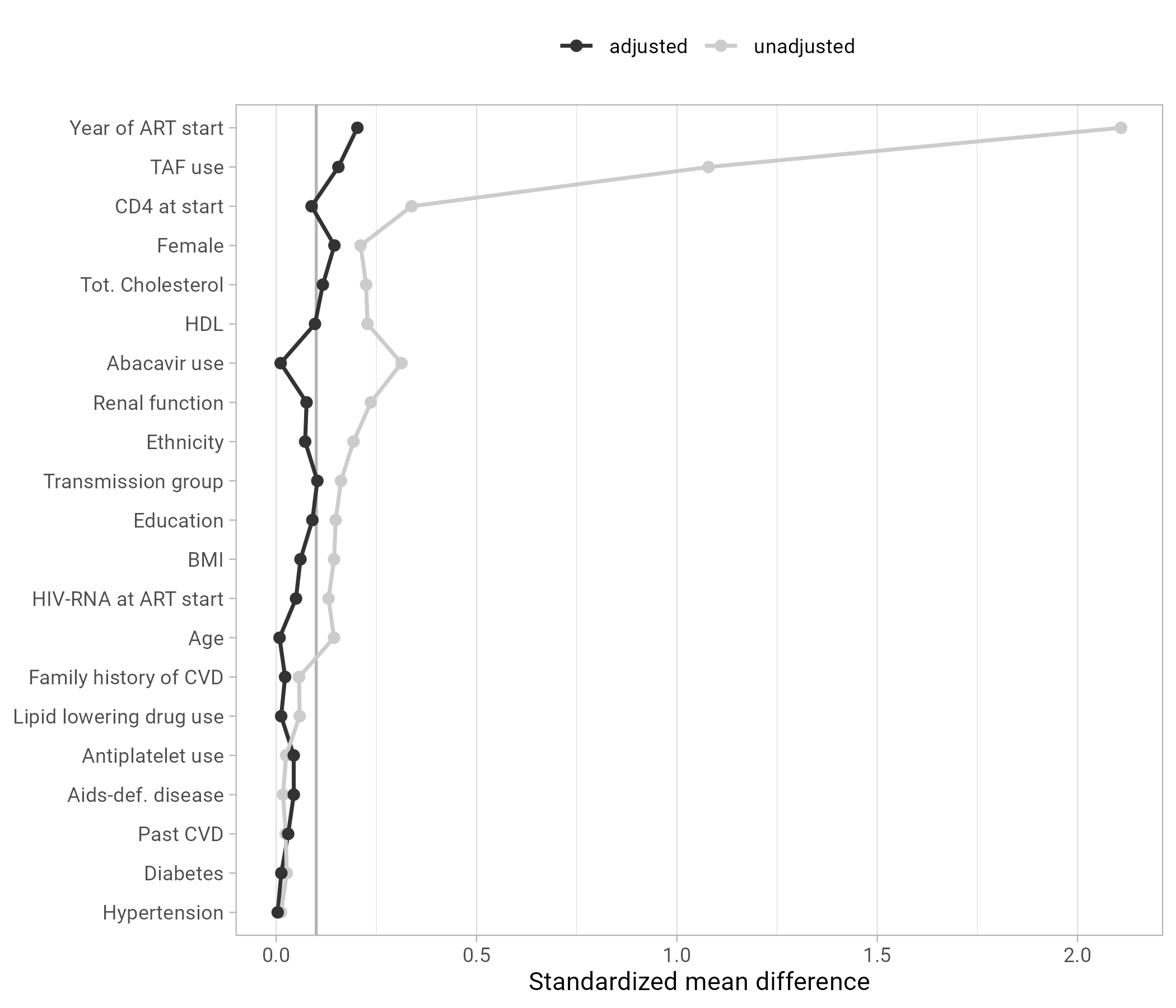


This plot shows the standardized mean difference (SDM) between covariates in the unadjusted (light grey) and in the inverse probability weighted population (black). They vertical grey line indicates a SDM of 0.1. Covariates below this line are considered to be well-balanced between the two groups. **ART** = antiretroviral therapy, **TAF =** tenofovir alafenamide, **HDL =** high densitiy lipoprotein, **BMI =** body mass index, **CVD =** cardiovascular disease.

**Figure S3** Sensitivity analysis restricted to individuals without history of cardiovascular disease at ART start (n = 5290)

**
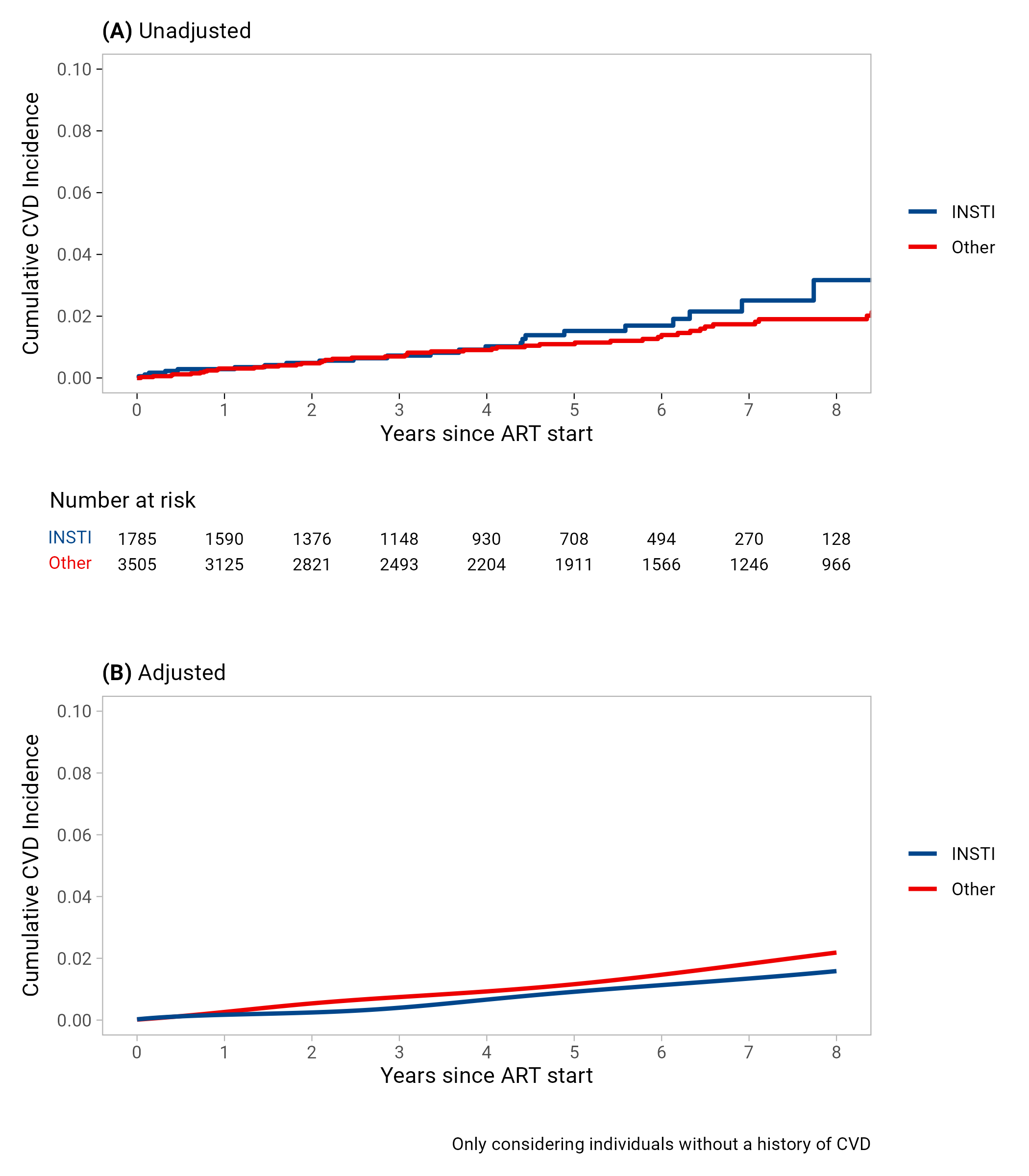
**

**Figure S4** Sensitivity analysis restricted to individuals who started ART after November 2011, when INSTI were recommended in European treatment guidelines (n = 3216)

**
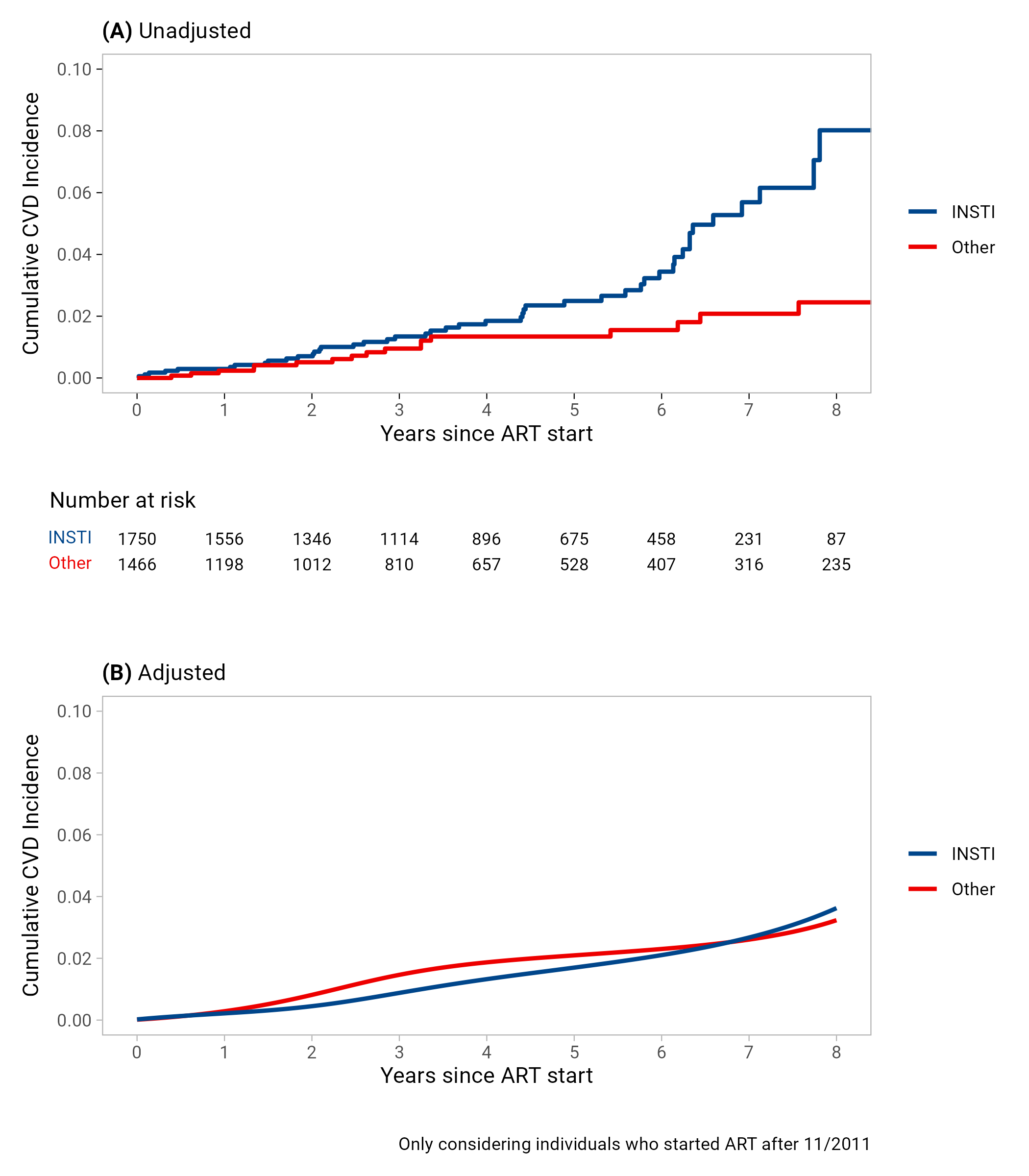
**

**Table S1** Patient characteristics at antiretroviral therapy start across follow-up periods

|  | **before 2012** | | **2012-2016** | | **after 2016** | |
| --- | --- | --- | --- | --- | --- | --- |
|  | **INSTI (N = 81)** | **Other (N = 2140)** | **INSTI (N = 809)** | **Other (N = 1270)** | **INSTI (N = 947)** | **Other (N = 115)** |
| **Female sex** | 10 (12%) | 519 (24%) | 135 (17%) | 300 (24%) | 146 (15%) | 35 (30%) |
| **Median age, years (IQR)** | 39 (31–45) | 38 (31–46) | 38 (31–49) | 37 (30–47) | 40 (31–50) | 36 (29–46) |
| **African origin** | 7 (8.6%) | 361 (17%) | 90 (11%) | 231 (18%) | 102 (11%) | 26 (23%) |
| **HIV transmission group** |  |  |  |  |  |  |
| MSM | 52 (64%) | 1,111 (52%) | 499 (62%) | 682 (54%) | 548 (58%) | 53 (46%) |
| Heterosexual contact | 21 (26%) | 780 (36%) | 234 (29%) | 448 (35%) | 295 (31%) | 41 (36%) |
| PWID | 7 (8.6%) | 136 (6.4%) | 34 (4.2%) | 57 (4.5%) | 33 (3.5%) | 3 (2.6%) |
| Other | 1 (1.2%) | 113 (5.3%) | 42 (5.2%) | 83 (6.5%) | 71 (7.5%) | 18 (16%) |
| **Highest education** |  |  |  |  |  |  |
| High-level education | 36 (44%) | 777 (36%) | 366 (45%) | 525 (42%) | 433 (46%) | 53 (46%) |
| Basic education | 41 (51%) | 1,203 (56%) | 388 (48%) | 657 (52%) | 445 (47%) | 55 (48%) |
| No prof. education | 4 (5%) | 141 (7%) | 48 (6%) | 72 (6%) | 48 (5%) | 6 (5%) |
| *missing* | *0* | *19 (1%)* | *7 (1%)* | *16 (1%)* | *21 (2%)* | *1 (1%)* |
| **AIDS-defining disease** | 10 (12%) | 257 (12%) | 64 (7.9%) | 122 (9.6%) | 122 (13%) | 16 (14%) |
| **Median CD4 nadir, cells/µL (IQR)** | 242 (204–328) | 266 (168–347) | 341 (214–479) | 308 (177–427) | 330 (158–504) | 324 (202–480) |
| **CD4 nadir** |  |  |  |  |  |  |
| ≥500 cells/µL | 3 (3.7%) | 126 (5.9%) | 157 (19%) | 159 (13%) | 200 (21%) | 15 (13%) |
| 350-499 cells/µL | 14 (17%) | 334 (16%) | 183 (23%) | 262 (21%) | 168 (18%) | 17 (15%) |
| 200-349 cells/µL | 37 (46%) | 831 (39%) | 194 (24%) | 316 (25%) | 180 (19%) | 19 (17%) |
| <200 cells/µL | 17 (21%) | 585 (27%) | 157 (19%) | 307 (24%) | 231 (24%) | 15 (13%) |
| *missing* | *10 (12%)* | *264 (12%)* | *118 (15%)* | *226 (18%)* | *168 (18%)* | *49 (43%)* |
| **HIV viral load at ART start** |  |  |  |  |  |  |
| 50-199 cp/mL | 7 (8.6%) | 142 (6.6%) | 28 (3.5%) | 67 (5.3%) | 52 (5.5%) | 8 (7.0%) |
| 200-100'000 cp/mL | 39 (48%) | 1,207 (56%) | 487 (60%) | 656 (52%) | 465 (49%) | 48 (42%) |
| >100'000 cp/mL | 28 (35%) | 589 (28%) | 224 (28%) | 374 (29%) | 342 (36%) | 23 (20%) |
| *missing* | *7 (8.6%)* | *202 (9.4%)* | *70 (8.7%)* | *173 (14%)* | *88 (9.3%)* | *36 (31%)* |
| **History of CVD** | 0 (0%) | 31 (1.4%) | 18 (2.2%) | 12 (0.9%) | 10 (1.1%) | 1 (0.9%) |
| **Family history of CVD** | 9 (11%) | 212 (9.9%) | 96 (12%) | 130 (10%) | 104 (11%) | 10 (8.7%) |
| **Diabetes** | 1 (1.2%) | 38 (1.8%) | 18 (2.2%) | 24 (1.9%) | 20 (2.1%) | 0 (0%) |
| **Arterial hypertension** | 14 (17%) | 240 (11%) | 84 (10%) | 100 (7.9%) | 91 (9.6%) | 10 (8.7%) |
| **Renal function** |  |  |  |  |  |  |
| ≥90 mL/min | 49 (60%) | 1,378 (64%) | 534 (66%) | 780 (61%) | 620 (65%) | 69 (60%) |
| 60-89 mL/min | 9 (11%) | 343 (16%) | 142 (18%) | 174 (14%) | 214 (23%) | 14 (12%) |
| <60 mL/min | 1 (1.2%) | 38 (1.8%) | 14 (1.7%) | 16 (1.3%) | 27 (2.9%) | 0 (0%) |
| *missing* | *22 (27%)* | *381 (18%)* | *119 (15%)* | *300 (24%)* | *86 (9.1%)* | *32 (28%)* |
| **Median eGFR, mL/min (IQR)** | 108 (96–116) | 106 (93–118) | 105 (92–116) | 107 (94–118) | 103 (88–115) | 108 (96–122) |
| **Smoking status** |  |  |  |  |  |  |
| current | 40 (49%) | 1,050 (49%) | 377 (47%) | 568 (45%) | 425 (45%) | 42 (37%) |
| past | 7 (8.6%) | 214 (10%) | 87 (11%) | 103 (8.1%) | 98 (10%) | 5 (4.3%) |
| never | 34 (42%) | 875 (41%) | 339 (42%) | 593 (47%) | 410 (43%) | 67 (58%) |
| *missing* | *0 (0%)* | *1 (<0.1%)* | *6 (0.7%)* | *6 (0.5%)* | *14 (1.5%)* | *1 (0.9%)* |
| **BMI category** |  |  |  |  |  |  |
| Normal (18.5-24.9 kg/m2) | 35 (43%) | 1,037 (48%) | 392 (48%) | 553 (44%) | 431 (46%) | 34 (30%) |
| Overweight (25-29.9 kg/m2) | 12 (15%) | 364 (17%) | 149 (18%) | 226 (18%) | 213 (22%) | 16 (14%) |
| Obese (≥30 kg/m2) | 4 (4.9%) | 107 (5.0%) | 47 (5.8%) | 62 (4.9%) | 70 (7.4%) | 9 (7.8%) |
| Underweight (<18.5 kg/m2) | 2 (2.5%) | 101 (4.7%) | 29 (3.6%) | 46 (3.6%) | 47 (5.0%) | 5 (4.3%) |
| *missing* | *28 (35%)* | *531 (25%)* | *192 (24%)* | *383 (30%)* | *186 (20%)* | *51 (44%)* |
| **Use of antiplatelet agent** | 0 (0%) | 40 (1.9%) | 14 (1.7%) | 11 (0.9%) | 20 (2.1%) | 3 (2.6%) |
| **Use of lipid-lowering drug** | 2 (2.5%) | 41 (1.9%) | 24 (3.0%) | 19 (1.5%) | 22 (2.3%) | 2 (1.7%) |
| **Use of abacavir** | 4 (4.9%) | 271 (13%) | 296 (37%) | 134 (11%) | 130 (14%) | 6 (5.2%) |
| **Use of tenofovir alafenamide** | 1 (1.2%) | 1 (<0.1%) | 22 (2.7%) | 4 (0.3%) | 707 (75%) | 43 (37%) |
| **IQR** = interquartile range, **INSTI** = integrase strand transfer inhibitor, **MSM** = men who have sex with men, **PWID** = persons who inject drugs,  **ART** = antiretroviral therapy, **CVD** = cardiovascular disease, **eGFR** = estimated glomerular filtration rate, **BMI** = body mass index. | | | | | | |

**Table S2** Distribution of follow-up, outcome events and censoring events by exposure group

|  | **INSTI-based ART** N = 1837 | **Other ART**  N = 3525 |
| --- | --- | --- |
| **Median follow-up, in years (IQR)** | 4.1 (2.1–6.2) | 5.5 (2.6–8.4) |
| **Cumulative number of CVD events** |  |  |
| 6 months | 5 | 4 |
| 1 years | 5 | 11 |
| 2 years | 11 | 17 |
| 5 years | 29 | 41 |
| 8 years | 45 | 57 |
| **Cardiovascular disease events** | 48 (2.6%) | 68 (1.9%) |
| Stroke | 11 (0.6%) | 25 (0.7%) |
| Myocardial infarction | 17 (0.9%) | 20 (0.6%) |
| Invasive cardiovascular procedure | 20 (1.1%) | 23 (0.6%) |
| **Artificial censoring events** |  |  |
| Stopped INSTI | 111 (6%) |  |
| Switched to INSTI |  | 2217 (62.9%) |
| **Loss to follow-up** | 244 (13%) | 512 (15%) |
| **IQR =** interquartile range**, INSTI =** integrase strand transfer inhibitor, **ART** = antiretroviral therapy | | |
